# E4orf1 protein reduces the need for endogenous insulin

**DOI:** 10.1038/s41387-019-0085-x

**Published:** 2019-05-24

**Authors:** Swetha Peddibhotla, Vijay Hegde, Md Akheruzzaman, Nikhil V. Dhurandhar

**Affiliations:** Department of Nutritional Sciences, Texas Tech University, Lubbock, TX USA

**Keywords:** Endocrinology, Type 2 diabetes

## Abstract

**Background:**

E4orf1 protein derived from adenovirus-36 reduces glucose excursion in mice, and lowers endogenous insulin response, suggesting a reduced need for insulin. We tested if the E4orf1-mediated lowering of insulin response is due to increased tissue sensitivity to insulin, reduced ability to produce or release insulin, or a reduced need for insulin release.

**Methods:**

*Experiment 1*: hyperinsulinemic–euglycemic clamps (HEC) and glucose tolerance test (GTT) were performed in high fat fed transgenic mice expressing E4orf1 or non-transgenic littermates (*n* = 12 each), for 4 weeks. *Experiments 2, 3, and 4*: E4orf1 or null vectors were expressed in rat-pancreatic β-cell line (INS-1) for 72 h, and cells were exposed to varying levels of glucose. Cell lysates and media were collected. *Experiment 5*: 3T3L1-preadipocytes that express E4orf1 upon doxycycline induction, or null vector were induced with doxycycline and then exposed to protein transport inhibitor. Supernatant and cell lysate were collected. *Experiment 6*: 3T3L1-preadipocytes that express E4orf1 upon doxycycline induction, or null vector were co-cultured with INS-1 cells for 24 h. Media was collected.

**Results:**

*Experiment 1*: E4orf1 transgenic mice cleared glucose faster compared to non-transgenic mice during GTT. HEC showed that E4orf1 did not alter tissue sensitivity to exogenous insulin in mice. *Experiments 2, 3, and 4*: in INS1 cells, E4orf1 did not alter Glut2 abundance or Akt activation, suggesting no reduction in glucose sensing or insulin synthesis, respectively. E4orf1 did not influence glucose-stimulated insulin secretion in media by INS1 cells. *Experiment 5*: E4orf1 was present in cell lysate, but not in media, indicating it is not a secretory protein. *Experiment 6*: INS1 cells released less insulin in media when co-cultured in the presence of E4orf1-expressing 3T3-L1 cells.

**Conclusions:**

Our studies support the working hypothesis that the E4orf1-mediated lowering of insulin response is not due to increased tissue sensitivity to insulin, or reduced ability to produce or release insulin, but likely to be due to a reduced need for insulin release.

## Introduction

The binding of insulin to the insulin receptor (IR) on the cell membrane is followed by tyrosine phosphorylation of insulin receptor substrates (IRS) 1 and 2. This proximal insulin signaling further leads to distal insulin signaling, comprising of the activation of phosphatidylinositol 3-kinase (PI3K), Akt, and translocation of glucose transporter 4 (Glut4) to the cell membrane, which facilitates the uptake of glucose in cells such as myocytes or adipocytes^1–3^. In obesity or type 2 diabetes (T2D), proximal insulin signaling is often impaired^4–6^. Yet, the actions of many anti-diabetic drugs such as the insulin mimetics, secretagogues, or sensitizers focus on recruiting the proximal insulin signaling for enhancing cellular glucose uptake. If the proximal insulin signaling is impaired, it would be harder to use these agents to increase cellular glucose uptake. Hence, an agent which promotes glucose disposal without recruiting proximal insulin signaling is likely to be more attractive for diabetes management.

Ad-36-specific E4orf1 is such a candidate. It is a 125-amino acid protein encoded by the E4orf1 (E4 open reading frame 1) gene of human adenoviruses^7^. E4orf1 increases glucose uptake in preadipocytes, adipocytes, myoblasts, and reduces glucose output from hepatocytes^8–13^ and improves glycemic control in mice^14–16^. Importantly, E4orf1 operates by activating the distal insulin signaling pathway and bypassing the IRS-mediated proximal insulin signaling pathway^7–10,17,18^. This makes E4orf1 a highly attractive potential anti-diabetic agent.

As a potential anti-diabetic agent, another attractive property of E4orf1 is its “insulin sparing action”^12^. Expression of E4orf1 in mice significantly reduces glucose excursion, compared to control mice without E4orf1 expression, and lowers the insulin response to glucose load, suggesting a reduced need for endogenous insulin^14–16,19^. Importantly, this reduced need for endogenous insulin, termed as the “*insulin sparing action*” may be exploited to help prevent pancreatic β cell damage, which is a common progression from insulin resistance E4orf1-based strategy might reduce hyperinsulinemia of insulin resistance and thereby, prevent or delay β cell failure. However, it is first necessary to precisely understand the underlying mechanism. We hypothesized that the reduction in circulating insulin response to glucose load in the presence of E4orf1 may either be due to (i) increased tissue sensitivity to insulin, (ii) pancreatic β cell damage by E4orf1, (iii) impaired insulin production or release, or (iv) reduced need for insulin release. Of these potential possibilities, increased tissue sensitivity to insulin is unlikely as E4orf1 in fact inhibits IRS1 and IRS2 signaling by phosphorylating their serine residue^11^. Moreover, insulin tolerance test in mice showed no greater sensitivity to exogenous insulin in the presence or absence of E4orf1^16^. Also, E4orf1 does not damage pancreatic β cells^16^. Nonetheless, this study focused on the key possibilities: (a) E4orf1 improves tissue sensitivity to insulin, (b) E4orf1 directly impairs insulin production or release, or (c) enhanced glucose clearance by E4orf1 indirectly reduces the stimulus to pancreatic β cells to release insulin.

Although for the above-stated reasons, E4orf1 was unlikely to increase insulin sensitivity, we tested it with hyperinsulinemic–euglycemic clamp study, which is the gold standard to determine tissue sensitivity to insulin^20^. The study requires maintaining a high insulin level by with exogenous insulin, while maintain a steady glucose level. The procedure quantifies tissue sensitivity to insulin, depending on the rate and amount of glucose needed to maintain euglycemia. Higher tissue sensitivity to insulin would require more glucose to maintain euglycemia.

The post-prandial rise in blood glucose is sensed by pancreatic β cells via the glucose transporter 2 (GLUT 2)-facilitated glucose diffusion into the cells^21,22^, which triggers insulin release^23^ by activating exocytosis of insulin-containing granules. Insulin self stimulates pro-insulin biosynthesis^24,25^ by binding to IR which phosphorylates tyrosine residue of IRS2 and triggers the activation of PI3K^26^, Akt and transcription factors involved in the expression of many other genes including insulin^26–29^.

Here, we used INS-1 cells (rat pancreatic β cells) to test if a direct presence of E4orf1 in cells interferes with glucose sensing (by interfering with Glut 2), or impairs the ability of the β cells to produce or release insulin in response to glucose exposure. Next, to test the indirect effect of E4orf1 on INS-1 cells, we first determined that E4orf1 is not a secretory protein. Next, a co-culture experiment tested if the enhanced glucose clearance from media by E4orf1-expressing 3T3-L1 cells (pTRE cells) indirectly reduces the need for the β cells (INS-1) to release insulin.

## Materials and methods

### Materials

#### Cell culture and AAV2 vectors

3T3-L1 preadipocytes were purchased from ATCC (#CL-173) and were maintained in DMEM without sodium pyruvate (Fisher Scientific, #MT10017CV) with 10% bovine calf serum (Fisher Scientific #SH3007203). INS-1 (Insulinoma cell line) cells were obtained from AddexBio (#C0018007) and maintained in INS-1 medium of optimized RPMI 1640 medium (AddexBio, #C0004-02) with 10% fetal bovine serum (Atlanta Biologicals, #S11150), 1% antibiotic–antimycotic agent (Sigma Aldrich, #A5955-100ML) and β-mercaptoethanol. The AAV2-E4orf1 and AAV2-Null (#SL100812) vectors were purchased from SignaGen Laboratories. Doxycycline (1000 ng/mL, Sigma, #D3072-1ML) inducible 3T3L1-pTRE null clones (pTRE-null) and 3T3L1-E4orf1 (pTRE-E4orf1) clones were generated as previously described^30^, and maintained in DMEM without sodium pyruvate (Fisher Scientific, #MT10017CV) Tet-free fetal bovine serum (Clonetech, #631101) with 0.25 µg/mL puromycin (Invivogen, #ant-pr-1) and 0.05 µg/mL hygromycin (Invitrogen, #10687-010).

The following three cell lines were used for conducting the experiments described below.

(A) 3T3-L1 cells were infected with either AAV2-Null or AAV2-E4orf1 to optimize the conditions for functional expression of E4orf1.
(B) INS-1 (rat pancreatic insulin secreting β-cell-derived cell line) cells were infected with E4orf1-expressing adeno-associated vector AAV2-E4orf1 (10^10^ GC) or control AAV2-Null (10^10^ GC). Seventy-two hours post-infection, cells were glucose starved in HBSS buffer for 2 h and stimulated with either 3 or 15 mM glucose for an additional 2 h. Experiments 2–4 and 6 were conducted as described below.
(C) 3T3-L1 cells that inducibly express E4orf1 in response to doxycycline (pTRE-3T3-L1-E4orf1) or expressing a null vector (pTRE-3T3-L1-Null)^30^ were used as described in “Techniques and assays”.

### Techniques and assays (experiments conducted)

#### Experiment 1: does E4orf1 expression improve tissue insulin sensitivity in high fat fed mice?

Institutional Animal Care and Use Committee (IACUC) of Texas Tech University approved the protocols for animal studies. Six–eight-week old E4orf1 transgenic male and female mice (*n* = 12) (described previously^14^) were obtained from our breeding colony and placed on a 12 h light–dark cycle at 25 °C and housed in micro-isolator cages, with ad libitum access to food and water. Mice were placed on a high fat (HF, 60% kcal) diet (Research Diets Inc. #D12492i) supplemented with 600 mg/kg doxycycline for 4 weeks to induce E4orf1 expression. Non-transgenic littermates (*n* = 12) were also placed on HF diet supplemented with 600 mg/kg doxycycline as control.

##### Glucose tolerance test (GTT)

Following transgene expression, GTT was performed to determine blood glucose clearance. Subsequent to a 4 h fast, conscious mice were injected with D-glucose (1.5 mg/kg of body weight) intra-peritoneally. Blood was collected from the tail vein prior to glucose injection (time 0) and at 15, 30, 60, and 120 min post-injection. Blood glucose was determined using a glucometer (AlphaTRAK 2 Veterinary Blood Glucose Monitoring System, #32107, ADW Diabetes).

##### Hyperinsulinemic–euglycemic clamps

The mice were then shipped to the Vanderbilt mouse metabolic phenotyping center to determine insulin sensitivity by hyperinsulinemic–euglycemic clamps as described previously^31^. Briefly, the clamp study used continuous infusion of 3H-Glucose to assess glucose turnover rates and continuous infusion of insulin at 2.5 mU/kg/min in 5 h fasted mice.

#### Experiment 2: does E4orf1 reduce glucose sensing by pancreatic β cells as indicated by Glut2 levels?

##### Flow cytometer

Glut2 expression was analyzed by flow cytometry. INS-1 cells were glucose starved for 2 h followed by stimulation with 15 mM glucose for 2 h. Following stimulation, cells were trypsinized and re-suspended in FACS buffer (PBS/3% FCS) containing Glut2 antibody (Bioss Antibodies, #bs-0351R) for 30 min at 4 °C. Cells were then washed and incubated with Alexa Fluor 488 for another 30 min at 4 °C, followed by washing 3× with 1× PBS and fixed in 2% PFA for 15 min at RT and stored in FACS buffer at 4 °C in dark. Fixed cells were analyzed using flow cytometer (Attune NxT, Thermo Fisher Scientific) and for each sample, 5000 cells were counted.

##### Western blotting

INS-1 cells were glucose starved for 2 h followed by glucose stimulation with either 3 or 15 mM glucose for 2 h. Cells were lysed in RIPA buffer (Enzo Life Sciences, #ADI-80-1496) with protease inhibitors (Sigma Aldrich, #592791001). Protein estimation was performed using Pierce BCA protein assay kit (Fisher Scientific, #PI23225). Protein lysates (30 µg) were separated by SDS-PAGE electrophoresis, transferred onto PVDF membrane and immunoblotted with Glut2 (1:250; Santa Cruz, #sc-9117), GAPDH (1:10,000; BioRad, #2118S).

#### Experiment 3: does E4orf1 affect insulin synthesis signaling in pancreatic β cells as indicated by Akt phosphorylation?

Western blotting was done as described above. The blot was probed with pAKt (1:250; Cell Signaling, #9271L) and then membrane was stripped and re-probed to detect total Akt (1:1000; Cell Signaling, #4691S) expression. The immunoblotted Akt levels were normalized to total Akt levels.

#### Experiment 4: does E4orf1 reduce insulin production or its release in pancreatic β-cells?

##### Glucose-stimulated insulin secretion assay

INS-1 cells (Null or E4orf1 groups) were glucose starved (3 mM glucose) for 2 h in HBSS buffer (114 mM NaCl, 4.7 mM KCl, 1.2 mM KH_2_PO_4_, 1.16 mM MgSO_4_, 20 mM HEPES, 2.5 mM CaCl_2_, 25.5 mM NaHCO_3_, 0.2% BSA) and then stimulated with 15 mM glucose for 2 h in HBSS buffer. The supernatant was centrifuged to remove any residual cells and analyzed for insulin levels using ELISA kit (Rat/Mouse Insulin ELISA, #EZRMI-13K, Millipore).

##### qRT-PCR

Total RNA from the Null- or E4orf1-vector-infected INS-1 cells was isolated using the RNeasy Plus Mini Kit (Qiagen). Complementary DNA was synthesized with the Maxima H Minus cDNA Synthesis Kit (Thermo Scientific) from 1 µg of RNA. Primers (5′-3′) for E4orf1 (Forward: gccccgaatctttccacatt, Reverse: tcccggcaaatatcacatcg) and GAPDH (Forward: GGTGAAGGTCGGTGTGAAC, Reverse: TGAGTGGAGTCATACTGGAACA) were designed for the qPCR reaction. The mRNA levels of the E4orf1 and GAPDH were determined by the Bio-Rad CFX RT-PCR detection system using the SYBR Green Supermix (Bio-Rad, #172-5121). GAPDH was used to normalize E4orf1 expression.

##### Immunofluorescence

Immunofluorescence analysis was performed with INS-1 cells infected with either Null or E4orf1 vector for 72 h on Lab-Tek Chamber slides (Nunc, #177402PK, Thermo Fisher Scientific). Cells were fixed in 2% PFA for 1 h at room temperature (RT), followed by rehydration in blocking buffer (phosphate-buffered saline [PBS]/10% FBS) for 1 h at RT. Following blocking, cells were incubated with 5 µg/mL of insulin antibody (Abcam, #ab63820) prepared in 1% BSA-PBST and kept overnight at 4 °C. Cells were washed with PBS and incubated with Alexa Fluor 647-conjugated, highly cross-adsorbed secondary antibodies (Jackson ImmunoResearch Laboratories Inc., #111-605-003) for 1 h at RT. The cells were washed 3 times and mounted in fluorescence mounting medium (ProLong® Diamond Antifade Mountant with DAPI, Thermo Fisher Scientific) and stored at 4 °C until viewed for image capture. Immunofluorescence microscopy was performed using an epifluorescence (Olympus BX-41) and confocal microscope (Olympus Fluoview FV300 Confocal Microscope).

#### Experiment 5: is E4orf1 a secreted protein?

##### E4orf1 secretion assay

Inducible pTRE-E4orf1 or pTRE-null cells at 70–80% confluency were induced with doxycycline (1000 ng/mL) for 6 h. The medium was replaced with 500 µl of pTRE media containing protein transport inhibitor cocktail (500×, Affymetrix eBioscience, #00-4980-03), either 1× (1 µl) or 5× (5 µl) and doxycycline. If we observed E4orf1 in media, it would imply E4orf1 is secreted by cells or it is due to cell lysis. We used protein transport inhibitor cocktail at a concentration of either 0, 1×, or 5× that would block the release of E4orf1 in media (to verify that the E4orf1 in media was secreted and not due to cell lysis). After 18 h, supernatant and trypsinized cells were collected and protein concentration was determined (Thermo Scientific™ Pierce™ BCA™ Protein Assay, Fisher Scientific, #PI23225). To immunoblot E4orf1 (1:250; Proteintech Inc.) by western blot analysis, 100 µg of supernatant and 30 µg of protein lysate was used. Western blotting was done as described in the section “Western blotting”.

Additionally, to ensure our ability to detect E4orf1 in media and to rule out the possibility of proteases in the supernatant that might inhibit detection of E4orf1 or degrade secreted E4orf1, supernatant from pTRE-E4orf1 and pTRE-null cells were mixed with different concentrations of purified cell lysate (10, 20, 30 µg) containing E4orf1 and immunoblotted for E4orf1.

#### Experiment 6: does E4orf1 indirectly reduce insulin secretion from β-cells by reducing glucose exposure?

##### Co-culturing

Transwell membrane filters (CLS3401-48EA, Sigma Aldrich) were placed in a 12-well tissue culture plate. Matrix for the filters was equilibrated with INS-1 medium (Optimized RPMI 1640/10% FBS/1% Pen-Strep/β-mercaptoethanol) overnight in a 37 °C incubator. Cells inducibly expressing E4orf1 (pTRE-E4orf1) or control cells (pTRE-null) were seeded on the matrix forming the upper layer, and INS-1 cells were seeded on the surface of the 12-well as the lower chamber. The two cell types were co-cultured for 24 h in serum-starved INS-1 medium containing doxycycline (1000 ng/mL) to induce E4orf1 expression. Cell growth medium (50 µl) was collected from the lower chamber at 0 and 24 h and stored at −20 °C. ELISA was performed to detect the levels of insulin.

## Results

### Experiment 1: E4orf1 expression does not enhance tissue insulin sensitivity in high fat fed mice

**Fig. 1:**
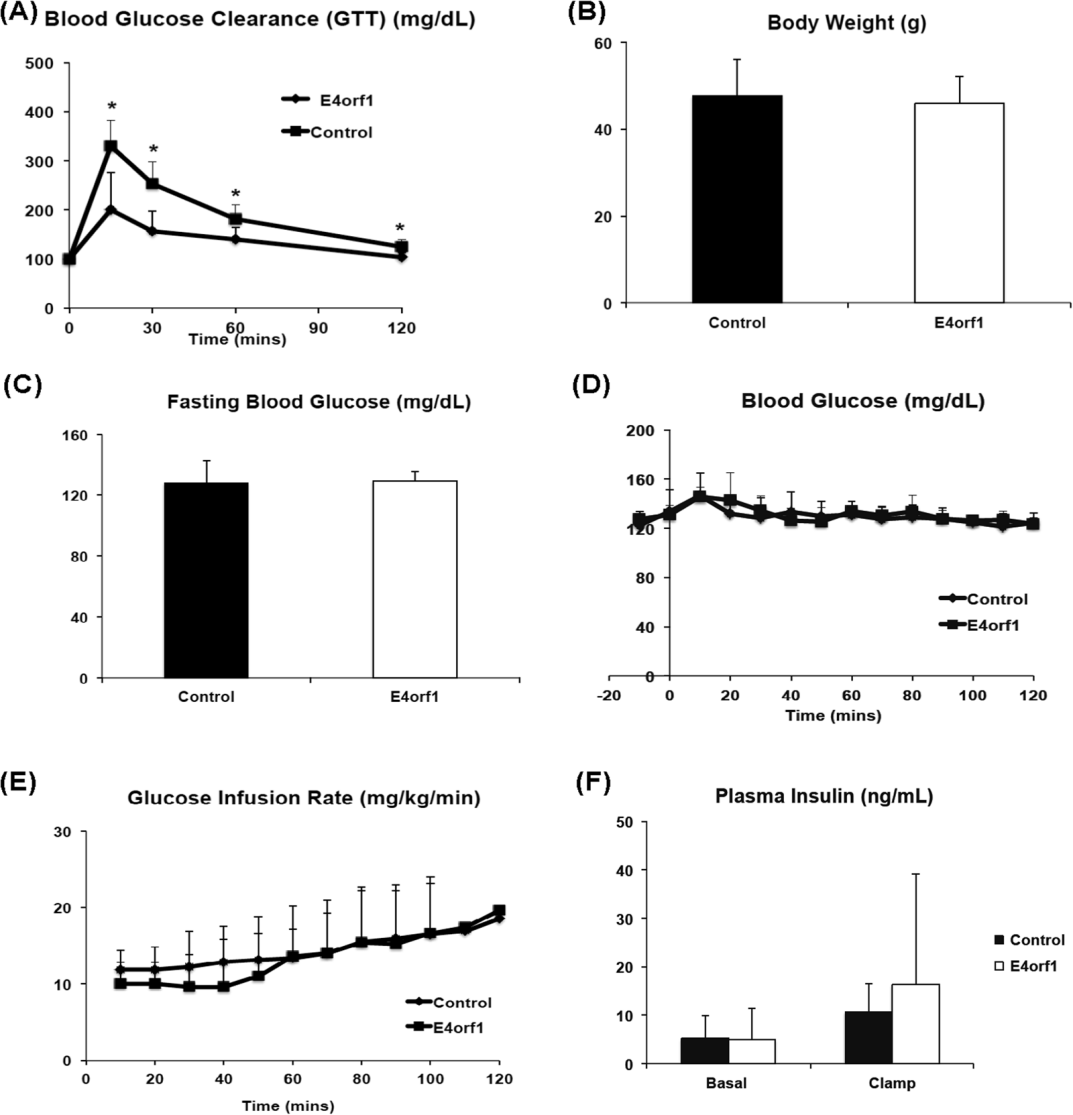
E4orf1 expression does not improve insulin sensitivity in high fat fed mice. Mean ± SD. **a** GTT was performed in E4orf1 transgenic and non-transgenic mice (after 4 h of starvation) by injecting D-glucose (1.5 mg/kg of body weight) intra-peritoneally. Blood glucose was determined at 0, 15, 30, 60, and 120 min post-glucose load shows significantly faster glucose clearance in E4orf1-expressing mice. **b** Body weight did not differ significantly between the two groups. The hyperinsulinemic–euglycemic clamp studies performed on these mice do not show any difference in **c** fasting blood glucose (mg/dL), **d** glucose infusion rate, or **e** plasma insulin (ng/mL). The groups were compared using *t*-test, *p ≤ 0.05

As expected, despite the high fat diet, E4orf1-expressing mice cleared blood glucose significantly faster than non-transgenic control mice (Fig. 1a). Prior to the hyperinsulinemic–euglycemic clamp studies, the two groups of mice had no significant difference in body weights (Fig. 1b). E4orf1 (*n* = 8) and control mice (*n* = 9) showed no difference in fasting blood glucose (Fig. 1c), euglycemia (Fig. 1d), glucose infusion rate (Fig. 1e), and plasma insulin (Fig. 1f), which was elevated from basal conditions, within groups during the clamp.

### Experiment 2: E4orf1 does not reduce glucose sensing by pancreatic β cells as indicated by Glut2 levels

**Fig. 2:**
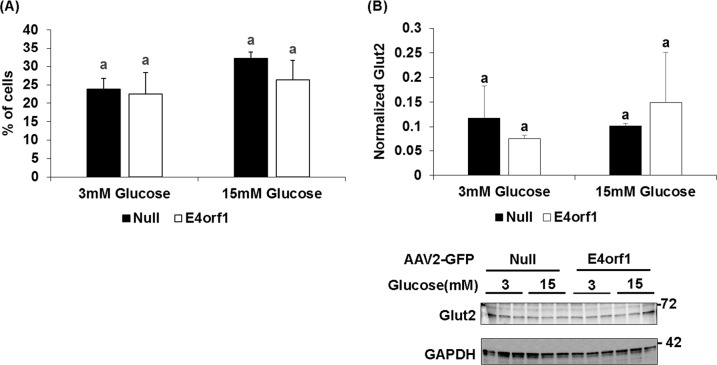
E4orf1 does not reduce glucose sensing by pancreatic β cells as indicated by Glut2 levels. INS-1 cells were infected with either Null or E4orf1 vector. 72 h post infection, cells were stimulated with either 3 or 15 mM glucose after glucose starvation. **a** INS-1 cells were stained with Glut2 antibody followed by Alexa Fluor 488 secondary antibody incubation to detect Glut2 by FACS analysis. **b** INS-1 cell lysates (30 µg) were immunoblotted for Glut2 and normalized to GAPDH. ImageJ was used to measure protein expression by densitometry. The groups were compared using ANOVA followed by Tukey’s test. Groups sharing letter are not statistically significantly different

Flow cytometry and western blot analysis determined Glut2 protein levels in INS-1 cells expressing either Null or E4orf1. Flow cytometry analysis showed no effect on levels of Glut2 protein abundance with or without E4orf1 (Fig. 2a). Upon glucose stimulation, expression of E4orf1 does not alter Glut2 levels (Glut2/GAPDH) as determined by western blotting (Fig. 2b). A lack of effect on Glut2 suggested that E4orf1 has no significant direct effect on glucose sensing in pancreatic β cells.

### Experiment 3: E4orf1 does not affect insulin synthesis signaling in pancreatic β cells as indicated by Akt phosphorylation

**Fig. 3:**
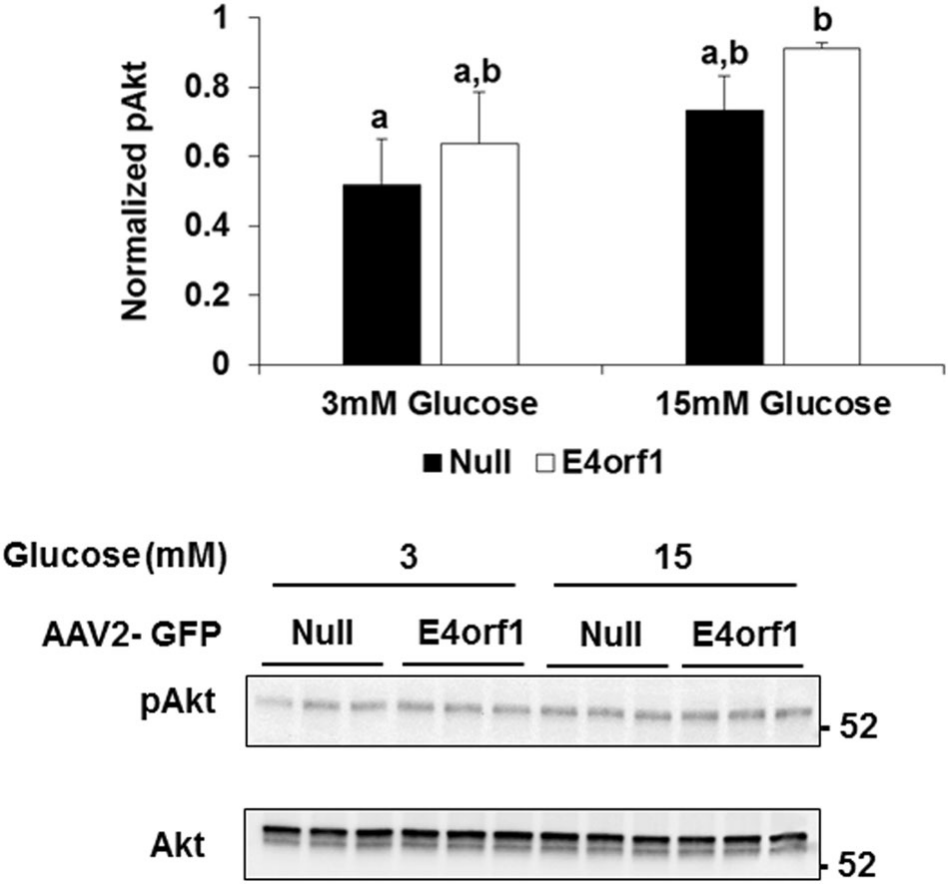
E4orf1 does not reduce signaling involved in insulin synthesis in pancreatic β cells as indicated by Akt phosphorylation. INS-1 cells were infected with either Null or E4orf1 vector. 72 h post infection and following glucose starvation, cells were stimulated with either 3 or 15 mM glucose. INS-1 cell lysates (30 µg) were immunoblotted with pAkt and normalized to total Akt. ImageJ was used to measure protein expression by densitometry. Groups were compared using ANOVA followed by Tukey’s test. Groups sharing letters are not statistically significantly different

Effect of E4orf1 expression on insulin synthesis in pancreatic β cells was determined by examining levels of Akt phosphorylation. INS-1 cell lysates expressing either null or E4orf1 vector did not show any differences in Akt phosphorylation levels (normalized to Akt) under conditions of glucose stimulation and E4orf1 expression (Fig. 3).

### Experiment 4: E4orf1 does not reduce insulin production or its release in pancreatic β cells

**Fig. 4:**
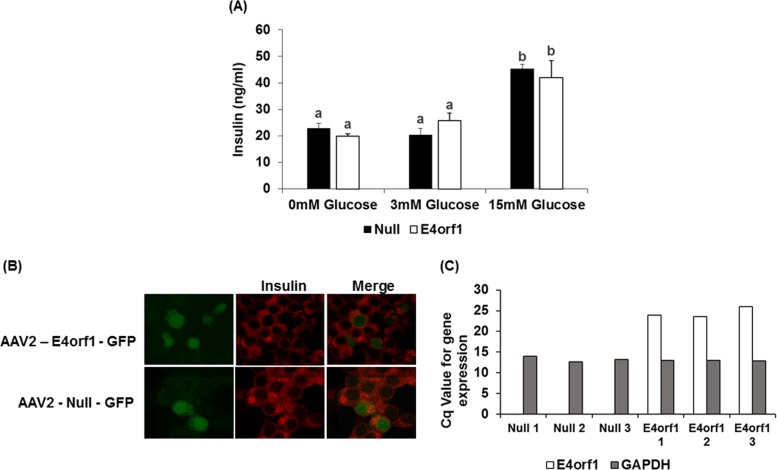
E4orf1 does not reduce insulin production or release in rat pancreatic β cells. INS-1 cells were infected with either Null or E4orf1 vector. 72 h post infection, cells were stimulated with either 0, 3, or 15 mM glucose following glucose starvation. **a** Supernatant was collected and concentration of insulin released was determined using ELISA. The groups were compared using ANOVA followed by Tukey’s test. Groups not sharing letters are statistically significantly different. **b** INS-1 cells were stained with antibody against insulin (red). Green autofluorescence indicates AAV2 presence. **c** E4orf1 expression in INS-1 cells infected with either Null (Null1, Null2, Null3, biological replicates) or E4orf1 vector (E4orf11, E4orf12, E4orf13, biological replicates) was tested using RT-PCR. *Y* axis represents Cq values for either E4orf1 or GAPDH expression. Gray bars represent GAPDH expression and black bars represent E4orf1 expression

To determine if E4orf1 reduced insulin release, insulin levels in media were determined by ELISA. As expected, INS-1 cells significantly increased insulin release upon glucose stimulation. However, there was no significant difference in insulin released by null or E4orf1 cells (Fig. 4a).

To confirm the expression of E4orf1, qRT-PCR was performed after RNA extraction and generation of cDNA from INS-1 cells infected with either null or E4orf1 vector. qRT-PCR showed expression of E4orf1 only in INS-1 infected with E4orf1 vector (Fig. 4c).

Insulin accumulation in β cells was determined by immunofluorescence staining of insulin in INS-1 cells with and without E4orf1 expression. The fluorescence intensity remained unaltered with and without E4orf1 expression (Fig. 4b).

### Experiment 5: E4orf1 is not a secreted protein

**Fig. 5:**
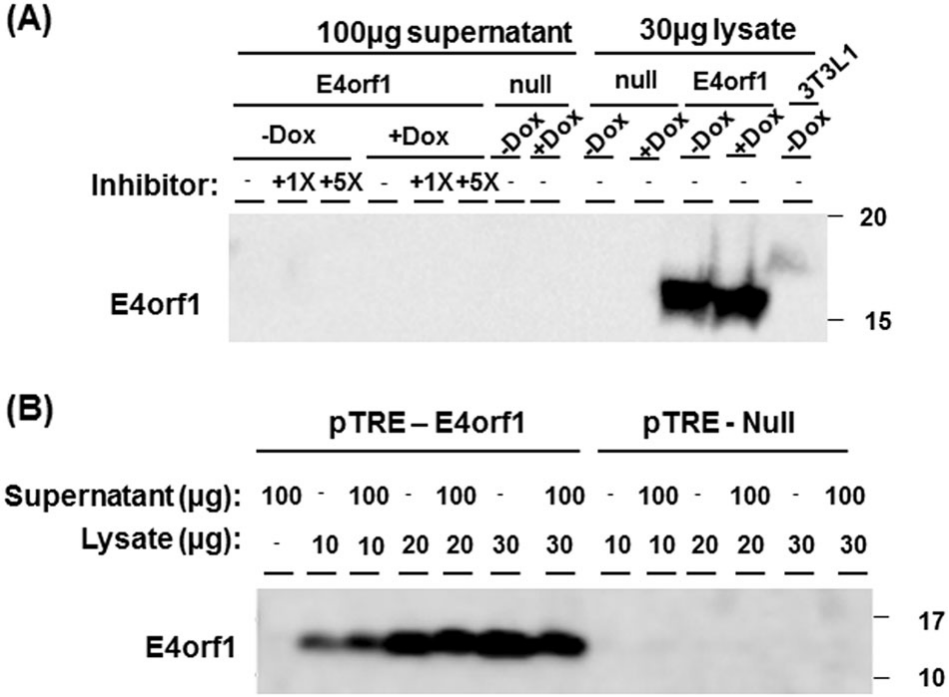
E4orf1 is not a secreted protein. **a** Both pTRE-null and pTRE-E4orf1 cells were treated with or without doxycycline (1000 ng/mL) and with 0× , 1×, or 5× protein transport inhibitor cocktail. Supernatant (100 µg) was immunoblotted to detect E4orf1. 30 µg of pTRE-E4orf1 (positive control) and pTRE-null (negative control) was used. **b** 100 µg of the pTRE-E4orf1 or pTRE-null cell supernatant was mixed with 10, 20, or 30 µg of E4orf1 or null lysate. Protein lysates were immunoblotted with E4orf1 antibody to determine E4orf1 expression by western blot analysis

To determine whether E4orf1 is a secreted protein, pTRE-E4orf1 cell supernatant and lysate was collected and western blot analysis performed to detect E4orf1. If E4orf1 was not detected in media, it would support that the protein is not secretory. If E4orf1 were detected in the media, it would be either due to release by cells, or due to cell lysis. Prior to collecting the supernatant, cells were also incubated with or without protein transport inhibitor cocktail. The protein transport inhibitor cocktail was added to block E4orf1 release, thereby distinguishing from cell lysis. E4orf1 was detected in cell lysate, but not in supernatants from pTRE-E4orf1 cells (Fig. 5a), suggesting that it is not a secretory protein.

We addressed if the absence of E4orf1 in the media is due to our inability to detect E4orf1 or possibly due to its protease-induced degradation. For this, the supernatant (100 µg) was supplemented with different concentrations of E4orf1 containing protein lysate (10, 20, and 30 µg) and immunoblotted for E4orf1. We could successfully detect E4orf1 when the supernatant was supplemented with E4orf1 lysate (Fig. 5b) but not in supernatant alone. Collectively, these results indicated that although E4orf1 was generated by the pTRE cells, it did not appear in the media.

### Experiment 6: E4orf1 lowers insulin secretion indirectly by promoting cellular glucose uptake

**Fig. 6:**
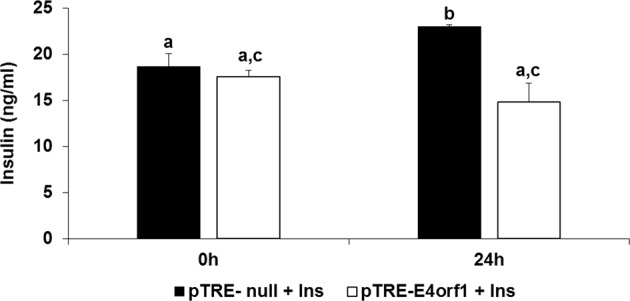
E4orf1 lowers insulin secretion indirectly by promoting cellular glucose uptake. INS-1 cells were co-cultured with either pTRE-null or pTRE-E4orf1 cells for 24 h. Concentration of insulin in media was detected using ELISA at 0 and 24 h. The groups were compared using ANOVA followed by Tukey’s test. Groups not sharing letters are statistically significantly different

INS-1 cells were co-cultured with either pTRE-null or pTRE-E4orf1 cells for 24 h in INS-1 serum-starved media (to avoid the confounding effects of insulin in serum). Insulin secretion in media was measured by ELISA. Insulin was significantly lower after 24 h co-culturing with pTRE-E4orf1 compared to pTRE-null cells (Fig. 6). Considering the ability of pTRE-E4orf1 cells to strongly upregulate cellular glucose uptake from media^30^, this observation indicates that cellular glucose uptake by E4orf1 reduces glucose stimulus to pancreatic β cells to release insulin.

## Discussion

E4orf1 gene of adenovirus Ad36 was identified as necessary and sufficient for the virus to improve glucose disposal in vitro and in vivo^32^. Independent of the virus, E4orf1 protein promotes glucose uptake in adipocytes, myoblasts and reduces glucose output from hepatocytes^8,9,11–13,17^, and in vivo, it improves high fat induced hyperglycemia, and reduces the response of endogenous insulin to glucose load^14–16^. Most importantly, E4orf1 promotes cellular glucose disposal in the absence of insulin^9^ or independent of the proximal insulin signaling^11,33^. This property is highly attractive for the potential use of E4orf1 as a drug to improve glycemic control in the presence of impaired proximal insulin signaling. Another desirable property of E4orf1 is its ability to reduce endogenous insulin levels in vivo^14–16^. Considering the adverse and complex health implications of elevated insulin levels due to insulin resistance^34,35^, a reduction in endogenous insulin response with concurrent improvement in glucose clearance is highly desirable.

A biochemical and cellular understanding of how E4orf1 reduces endogenous insulin response to glucose load should provide important new insight into the regulation of insulin metabolism. Our working hypothesis was that E4orf1 promotes glucose disposal independent of insulin or insulin signaling, which reduces the need for endogenous insulin response to a glucose load. To be comprehensive, we tested if the reduction in endogenous insulin response to glucose load in the presence of E4orf1 is due to greater tissue sensitivity to insulin, or reduced ability to produce or release insulin, or a reduced need for insulin release.

While earlier evidence had suggested that E4orf1 does not increase tissue sensitivity to insulin^11,16^ here we used the gold standard test to determine insulin sensitivity. Hyperinsulinemic–euglycemic clamp study showed that insulin sensitivity between E4orf1-expressing and control groups was not significantly different. This led us to test a direct or indirect effect of E4orf1 on pancreatic β cells by investigating the changes in cell signaling indicators of glucose sensing or insulin synthesis or release. We investigated whether E4orf1 attenuates the glucose sensing ability of pancreatic β cells by lowering Glut2 levels, or reducing Akt activation, thereby lowers insulin synthesis or its release from the β cells.

Glut2 is central to glucose sensing by the β cells^36^. Also, self-stimulated pro-insulin biosynthesis by insulin involves the activation of PI3K^26^ which leads to Akt phosphorylation and the eventual expression of insulin gene expression^26–29^. When INS-1 cells were infected with AAV2 vectors, the expression of E4orf1 in cells revealed the direct effect of E4orf1 on INS1 cells. We observed no change in Glut2 levels or pAkt abundance in β cells due to E4orf1, suggesting that glucose sensing or insulin synthesis are not compromised as a direct effect of E4orf1. The fact that E4orf1 does not influence insulin synthesis was also supported by immunostaining of insulin of the β-cells. Moreover, E4orf1 does not block the release of insulin. As expected, the β cells responded to increased glucose in media, by releasing more insulin. This response was not influenced by the presence of E4orf1 in cells. These studies indicated that E4orf1 does not directly affect insulin synthesis or release from pancreatic β cells. The findings are consistent with our previous findings that E4orf1 does not negatively effect β cell morphology^14,16^.

Next, we tested the indirect effect, if any, of E4orf1 on insulin release by β cells. For this, we confirmed that E4orf1 is not a secreted protein. So, in co-culture with cells expressing E4orf1, there would be no direct effect of E4orf1 secreted in the media. We hypothesized that lower endogenous insulin response in presence of E4orf1 is due to reduced need for insulin. pTRE cells expressing E4orf1 show substantial glucose uptake into the cells from media^30^. Hence, we infer that lower glucose level in the media reduces stimulus to β cells, which in turn reduces insulin response.

In summary, the reduction in endogenous insulin response in the presence of E4orf1 seems unlikely due to greater insulin sensitivity, or a reduction in the ability of the β cells to synthesize or release insulin. Instead, it is more likely due to the *insulin sparing action* resulting from E4orf1-induced glucose clearance.

Circumstances when the systemic endogenous insulin response could be lower, include insulin insufficiency (e.g., type 1 diabetes), or high insulin sensitivity which reduces insulin requirement. This study provides a third possibility described by a unique model. E4orf1 reduces endogenous insulin requirement without increasing its sensitivity. However, besides glucose disposal, insulin also influences other key metabolic functions such as lipolysis, lipogenesis, and protein metabolism. Those functions may be affected by lower endogenous insulin amount, especially when insulin sensitivity has not improved. This suggests that while E4orf1 enhances glucose disposal, the reduction in endogenous insulin may modify lipogenesis and lipolysis. Next studies will be aimed at addressing these queries.

Overall, E4orf1 offers a novel template with unique properties to develop antihyperglycemic drugs to treat insulin resistance, and diabetes. Future studies are needed to investigate if E4orf1-induced reduction in endogenous insulin response will help reduce pancreatic β cell damage that often follows insulin resistance.

## Supplementary information

Supplemental Material
