# Supplementary material for: E4orf1 protein reduces the need for endogenous insulin
Source: Nutr Diabetes. 2019 May 24;9:17. doi: 10.1038/s41387-019-0085-x (PMC6534626; doi:10.1038/s41387-019-0085-x)
Supplement: Supplementary file 1 — Supplemental Material [file 41387_2019_85_MOESM1_ESM.doc]

**Supplementary information**

**Materials and Methods**

**Characterization of E4orf1-expressing Adenovirus Associated Vectors (AAV)**

1. To determine the role and effect of E4orf1, we generated E4orf1-expressing AAV. As an initial screen for the right vector serotype, infection and expression efficiency of commercially available AAV serotypes 2 and 9 (SignaGen Labs, Gaithersburg, MD) were tested in murine 3T3-L1 preadipocytes. These AAV vectors have a GFP fluorescent tag, which allow visualization of infection efficiency and expression over time. Serotype2 showed better infection efficiency and expression compared to AAV9 (data not shown). AAV2 expressing E4orf1 was custom made by SignaGen labs by inserting purified E4orf1 plasmid DNA into the AAV2 serotype. To determine the duration of GFP expression by the recombinant AAV2-E4orf1, murine 3T3-L1 cells were infected with a serial dilution (1×10^11^GC/mL-1×10^8^GC/mL) of the AAV2-E4orf1 viral stock (1×10^12^GC/mL). GFP fluorescence was observed for 15 days.
2. To determine the effect of recombinant AAV2-E4orf1, 3T3-L1 preadipocytes were infected with either null or E4orf1 vector (1×10^10^GC/mL-1×10^8^GC/mL). Glucose uptake was determined with 2 h serum starved cells after 4-days and 8-days of infection.

**Glucose uptake assay:**

Treated and serum starved cells were washed twice with 1X PBS followed by addition of Krebs-Ringer phosphate (KRP) buffer (900 µL) with 0.2% BSA [9]. For insulin stimulation, 100 nM insulin (Sigma # I0516) was supplemented in KRP buffer and cells were incubated for 20 min. To determine non-specific glucose uptake, cells were treated with 100 nM cytochalasin B (Sigma Aldrich #6762). Following incubation for 20 min, 100 µL of 10X isotope solution was added to each well for a final concentration of 100 nM cold 2-deoxy glucose (Sigma # D6134) and 0.5 mCi/mL [3H]- 2-Deoxyglucose (PerkinElmer #NEC720A250UC) for 5 min. Cells were immediately washed in ice cold 1X PBS (3X) at the end of 5 min and plates air-dried. To lyse cells, 1 mL of 0.05% SDS was added to each well and incubated at 37°C for 45-60 min. Protein lysates (900 µL) were added to individual scintillation vials containing 3 mL of scintillation liquid (Wheaton #986540) and 50 µL of sample used for protein estimation by bicinchoninic acid (BCA) assay. Radiolabeled glucose in cell lysate was measured using a Beckman scintillation counter (Perkin Elmer TriCarb 4810TR) and scintillation counts per minute were normalized to protein content of each well.

**Results: E4orf1 expression in 3T3L1 preadipocytes increases glucose uptake.**

The initial GFP expression of AAV2- Null and AAV2- E4orf1 was observed 4 d post-infection (data not shown) and appeared to increase until day 12 (Figure A) following which a decrease in expression was observed around day 15 (Figure A). Four days post infection, AAV2-E4orf1 (1×10^10^GC/mL and 1×10^8^GC/mL diluted vector) infected cells showed a significant increase in glucose uptake compared to AAV2-Null cells (Figure B). Eight days post-infection, compared to the AAV2-Null vector infected cells, AAV2-E4orf1 (1×10^10^GC/mL and 1×10^9^GC/mL diluted vector) showed significant increase in glucose uptake with (Figure C). These data show that the AAV2-vectors successfully deliver E4orf1 to cells, where it can promote glucose disposal.

**(A)**

x

**Figure Legend: AAV2 – E4orf1 infection and expression in 3T3L1 showed an increase in glucose uptake. (A)** 3T3L1 were infected with AAV2 –E4orf1 at different dilutions (1×10^11^GC/mL-1×10^8^GC/mL) and GFP fluorescence was observed for 15 days. **(B) and (C)** Glucose uptake was performed with 3T3L1 that were infected with either AAV2- null or AAV2-E4orf1 at different dilutions 4 days **(B)** and 8 days **(C)** post infection. AAV2-null and AAV2-E4orf1 groups were compared using t-test.
